# Alterations in Fetal Doppler Parameters Before and Twenty-Four Hours After Radiofrequency Ablation for Twin Reversed Arterial Perfusion Sequence

**DOI:** 10.3389/fmed.2022.802666

**Published:** 2022-04-14

**Authors:** Lan Zhang, Hongli Liu, Shuai Huang, Chao Tong, Zhigang Wang, Hongbo Qi, Philip N. Baker, Mark D. Kilby

**Affiliations:** ^1^State Key Laboratory of Maternal and Fetal Medicine of Chongqing Municipality, First Affiliated Hospital of Chongqing Medical University, Chongqing, China; ^2^Department of Obstetrics and Gynecology, First Affiliated Hospital of Chongqing Medical University, Chongqing, China; ^3^Fetal Medicine Unit, First Affiliated Hospital of Chongqing Medical University, Chongqing, China; ^4^International Collaborative Joint Laboratory of Reproduction and Development of Ministry of Education P.R.C., Chongqing Medical University, Chongqing, China; ^5^Institute of Ultrasound Imaging, Department of Ultrasound, Second Affiliated Hospital of Chongqing Medical University, Chongqing Key Laboratory of Ultrasound Molecular Imaging, Chongqing, China; ^6^College of Life Sciences, University of Leicester, Leicester, United Kingdom; ^7^Institute of Metabolism and System Research, University of Birmingham, Birmingham, United Kingdom

**Keywords:** middle cerebral artery, umbilical artery, doppler, twin reversed artery perfusion sequence, radiofrequency ablation, monochorionic twin

## Abstract

**Objective:**

To evaluate alterations in the fetal Doppler parameters of pump fetuses before and 24 h after radiofrequency ablation surgery for twin reversed arterial perfusion sequence (TRAPs).

**Methods:**

This is a retrospective study of 28 pump fetuses in TRAPs and 28 normal control twins between 2016 and 2021. The fetal Doppler parameters, including the umbilical artery pulsatility index (UA-PI), middle cerebral artery peak systolic velocity (MCA-PSV), middle cerebral artery pulsatility index (MCA-PI), and cerebroplacental ratio (CPR), of the controls, and pump fetuses before and 24 h after surgery were compared.

**Results:**

An increasing trend and a further increase in the MCA-PSV, MCA-PI, MCA-PSV Z score, and MCA-PI Z score after surgery were observed in pump fetuses with gestational age (GA) ≥20 weeks; however, such changes were not observed in those with a GA of <20 weeks. The UA-PI and CPR before and after surgery were not different between control and pump fetuses, whether the GA was ≥20 or <20 weeks.

**Conclusion:**

In the middle second trimester, the pump fetus might suffer from high cardiac output rather than hypoxemia before surgery and congestive heart failure, or hemodilutional anemia after surgery. This may provide some theoretical evidence in favor of early intervention, rather than waiting for a more advanced GA, to avoid unnecessary hemodynamic alterations.

## Introduction

Twin arterial reverse perfusion sequence (TRAPs) is a rare complication that is unique to monochorionic twin pregnancies. A recent study based on mathematical models reported that its morbidity is 2.6% in monochorionic twins and it affects 1 in 9,500 to 11,000 pregnancies (1). This complication is typified by a twin with an absent or rudimentary heart (acardiac fetus) that is oppositely perfused by its cotwin (pump fetus) *via* a large artery anastomosis in a single placenta (2). Intrafetal radiofrequency ablation (RFA) surgery has been shown to be effective in arresting reversed flows, thereby preventing the ongoing hemodynamic burden for the pump fetus (3, 4).

Vascular anastomoses are omnipresent in monochorionic placentae; hence, fetal hemodynamic disorders resulting from an intertwined blood transfusion are often present in complicated monochorionic twin pregnancies (5–7). Due to continuous and massive blood transfusion to its cotwin through large placental anastomoses, the pump fetus is reasonably thought to be in a state of high cardiac output. It has long been known that various cardiac and extracardiac conditions, such as fetal growth restriction, fetal tumors, a twin-twin transfusion syndrome, fetal anemia, arteriovenous fistula with high cardiac output, and congenital heart diseases, can alter the hemodynamic status and fetal cardiac function (8). In TRAPs, a significant proportion of survival pump twins develop congestive heart failure/chronic high-output failure if untreated (9).

Fetal hemodynamic disturbances and cardiac dysfunction may result in Doppler alterations (10, 11). Fetal Doppler is the most common and direct method to evaluate fetal safety *in utero*. The most commonly used fetal Doppler indicators include the umbilical artery pulsatility index (UA-PI), middle cerebral artery peak systolic velocity (MCA-PSV), middle cerebral artery pulsatility index (MCA-PI), and cerebroplacental ratio (CPR) (12, 13). The MCA-PSV is widely used to screen for fetal anemia (14). The UA-PI, a recognized indicator for placental resistance, can reflect the degree of fetal hypoxia when combined with the MCA-PI (15). The CPR is calculated as the ratio of MCA-PI to UA-PI. As shown by numerous studies, a lower CPR is a marker of a “brain-sparing” effect resulting from fetal hypoxemia (16, 17).

To date, there have been several related investigations evaluating Doppler alterations before and after surgery in women with twin-twin transfusion syndrome, which has a certain value for evaluating fetal prognosis (18, 19). However, to the best of our knowledge, it is unknown whether the pump fetus has Doppler abnormalities and whether the Doppler parameter changes after surgery for TRAPs in a short period, and this knowledge could help to evaluate surgical efficacy. Therefore, we aimed to assess alterations in the Doppler parameters of the pump fetus before and 24 h after RFA surgery for TRAPs. We hypothesized that the pump fetus might suffer from an underlying hemodynamic disorder, followed by an abnormal Doppler presentation, and that the alleviation of hemodynamic burden may provoke a hemodynamic response. This could provide a theoretical basis for the timing of treatment and understanding of the disease evolution after surgery.

## Materials and Methods

### Patients

This was a retrospective study of twin pregnancies with TRAPs at a tertiary referral center of the First Affiliated Hospital of Chongqing Medical University in Chongqing, China, between July 2016 and 2021. As this was a retrospective analysis of routinely collected anonymized clinical data, the local ethics committee confirmed that no ethical approval from the patients was necessary for accordance with national regulations.

The pregnancies with TRAPs at our center were enrolled as the TRAPs group. Normal monochorionic diamniotic twin pregnancies, which were matched to those in the TRAPs group for maternal age and gestational age (GA), as calculated by ultrasound, were randomly selected during the last 6 months of the study and enrolled as the normal control group. All fetuses were further divided into two subgroups according to whether GA at evaluation of MCA-PSV or RFA surgery was above 20 weeks. The inclusion criteria for the TRAPs group were pregnancies that underwent RFA surgery and Doppler evaluation before and 24 h after surgery. The diagnosis of TRAPs was based on characteristic ultrasonographic findings of a grossly malformed twin perfused with reversed umbilical flow from a normally formed twin in a monochorionic twin pregnancy. Based on the morphological characteristics, the acardiac fetuses were classified into four types: acardiac acephalic, acardiac anceps, acardiac acormus, and acardiac amorphous (20). The exclusion criteria were fetal loss related to preterm premature rupture of membranes or placental bleeding/abruption <1 week after RFA surgery; abnormality, aneuploidy, or a genetic syndrome of the pump fetus; and a higher-order multiple pregnancies. Maternal medical records, pre- and postoperative ultrasound findings, and details of the procedure were reviewed.

### Ultrasonic Evaluation

Voluson E8 and E10 ultrasound instruments (GE Healthcare, Austria) were used with C4-8-D (4–8 MHz) and C1-5-D (1–5 MHz) transducers. Complete ultrasound examinations were performed by an experienced sonographer (LZ), according to the International Society of Ultrasound in Obstetrics and Gynecology Practice guidelines (2). The Doppler values, including UA-PI, MCA-PSV, MCA-PI, and CPR, of the fetus nearer to the cervix in the control group, and the pump fetus before and 24 h after surgery were recorded. To correct for the influence of GA on Doppler values, the Z scores of UA-PI, MCA-PI, MCA-PSV, and CPR with a GA of ≥20 weeks were calculated; this process was based on the analysis of data derived from routine second- and third-trimester screening studies conducted by the fetal medicine foundation.^1^ The fetal weight was evaluated according to head circumference, biparietal diameter, abdominal circumference, and femur length using the Hadlock formula (21). The GA was determined according to the last menstrual period, the crown-rump length of the pump fetus at the ultrasound scan at 11^+0^–13^+6^ weeks, or the head circumference of the pump fetus when the first ultrasound scan was performed after 14 weeks of gestation. Chorionicity was determined by the number of placentae and the presence of a T-sign or a lambda sign. Amnionicity was determined by the presence of an intertwined membrane or cord entanglement.

### Radiofrequency Ablation Therapy

The RFA was performed by an experienced surgeon (SH) to block the flow within the acardiac fetus under local anesthesia. Under the guidance of real-time ultrasound, the percutaneous intrauterine intervention was performed using a 15-gauge probe and radiofrequency generator (Medsphere International Corporation, Beijing, China). To determine whether the procedures were successful, the power Doppler was used to confirm the absence of blood flow within the acardiac fetus.

### Statistical Analysis

The normality of the data was determined with the Shapiro–Wilk test. Continuous variables are expressed as the means ± standard deviations (SD) or as medians (interquartile ranges) as appropriate. Continuous data between the TRAPs group and the control group were compared by independent *T*-test or Mann–Whitney U test as appropriate. Pairwise comparisons of fetal Doppler values among the controls and the pump fetuses, before and after surgery, were carried out by related samples of Friedman’s two-way analysis. Categorical variables were expressed as numbers of cases and percentages and compared by Pearson’s chi-square test. All *p*-values were two-sided, and values of <0.05 were considered statistically significant. Statistical analyses were performed using SPSS version 21.0 (IBM Corporation, Armonk, NY, United States).

## Results

### General Information

**FIGURE 1:**
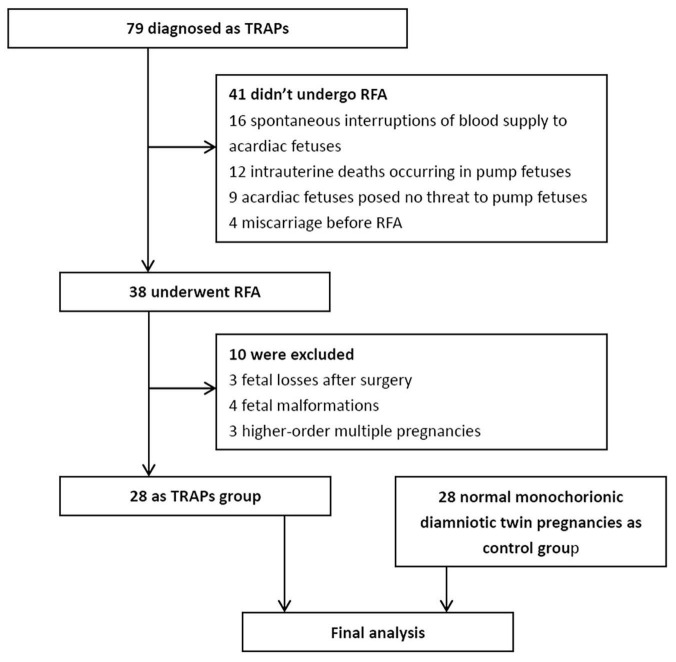
Study flow chart. TRAPs, twin arterial reversed perfusion sequence; RFA, radiofrequency ablation.

A total of 79 consecutive monochorionic pregnancies were diagnosed as TRAPs between July 2016 and 2021. Forty-one pregnancies with TRAPs did not undergo RFA surgery because 16 acardiac fetuses had a spontaneous interruption of blood supply, 12 pump fetuses experienced intrauterine death, 9 acardiac fetuses were not considered a threat to the pump fetuses, and 4 suffered from a miscarriage before RFA surgery. Thirty-eight pregnant women underwent RFA surgery. However, 3 had fetal loss after surgery, 4 had fetal malformations revealed by subsequent ultrasonography, and 3 had higher-order multiple pregnancies. Therefore, they were excluded. Finally, 28 pregnancies were enrolled in the TRAPs group in the final analysis. Additionally, 28 normal monochorionic diamniotic twin pregnancies matched for maternal age and GA were enrolled as the control group (Figure 1). In the TRAPs group, 2 pump fetuses had signs of heart failure (including right atrial dilation, tricuspid regurgitation, and pericardial effusion) before surgery, without significant postoperative relief, but both gradually returned to normal before delivery. New pericardial effusion occurred within 24 h after operation in 1 fetus but disappeared 2 days later. Twenty-four pregnancies were identified as monochorionic diamniotic twin pregnancies, and 4 were monochorionic monoamniotic twin pregnancies. According to the morphological characteristics of the acardiac fetuses, 24 fetuses were classified as acardiac acephalic, 4 were acardiac anceps, and none were acardiac acormus or acardiac amorphous.

### Clinical Characteristics

The clinical characteristics of the control group and the TRAPs group are presented in Table 1. There was a lower cesarean section rate, a later delivery GA, and a higher neonatal birth weight in the pump fetuses in the TRAPs group than in the fetuses in the control group (all *P* < 0.05). No significant differences in maternal age, pre-pregnancy body mass index, parity, evaluated fetal weight, or neonatal sex was found between the two groups (all *P* > 0.05).

**TABLE 1:** Clinical characteristics of the control group and the twin reversed arterial perfusion sequences (TRAPs) group.

Clinical characteristics	TRAPs (*n* = 28)	Controls (*n* = 28)	*P*-value
Maternal age (year)	28.54 ± 4.83	28.61 ± 4.62	0.955
Prepregnancy body mass index	21.19 ± 1.61	21.32 ± 1.43	0.753
Nulliparous, n (%)	20 (71.43%)	19 (67.86%)	0.771
Evaluated fetal weight (g)	271 (369)	284 (308)	0.902
Delivery GA (week)	37.65 (3.50)	36.07 (1.72)	0.029
Cesarean section, n (%)	11 (39.29%)	26 (92.86%)	<0.001
Neonatal birth weight (g)	3075 (1020)	2520 (528)	0.017
Male, n (%)	12 (42.86%)	15 (53.57%)	0.422

*TRAPs, twin reversed artery perfusion sequence; GA, gestational age. Values are given as the means ± standard deviations (SD) or as medians (interquartile ranges) or numbers (percentages).*

### Surgical Characteristics

The surgical characteristics of the two groups are detailed in Table 2. The median time of RFA surgery for the TRAPs group was 19.50 (15–25) weeks. Twelve surgeries were performed before 20 weeks, and 16 were performed after 20 weeks. Nine had to penetrate the placentae. Nine patients had 1 cycle of RFA coagulation, 19 had 2 cycles, and 1 had 3 cycles. The duration of RFA coagulation was 5.80 ± 2.09 min. The median total operative time was 45 (35–81) min. There were no differences in RFA cycles, duration of coagulation, or total operative time between fetuses with a GA of ≥20 weeks and fetuses with a GA of <20 weeks (all *P* > 0.05).

**TABLE 2:** Surgical characteristics.

Surgical characteristics	15 W∼25 W (*n* = 28)	<20 W (*n* = 12)	≥20 W (*n* = 16)	*P*-value (≥20 W vs. <20 W)
Number of RFA cycles				0.606
1, n (%)	8 (28.57%)	3 (10.71%)	5 (17.86%)	
2, n (%)	19 (67.86%)	9 (32.14%)	10 (35.71%)	
3, n (%)	1 (3.57%)	0 (0%)	1 (3.57%)	
Duration of RFA coagulation (min)	5.80 ± 2.09	5.56 ± 1.78	5.99 ± 2.33	0.599
Total operative time (min)	45.00 (33.25)	47.67 ± 21.32	50.06 ± 16.96	0.743

*RFA, radiofrequency ablation; W, weeks. Values are given as the means ± standard deviations (SD) or as medians (interquartile ranges) or numbers (percentages).*

### Doppler Alterations Before and 24 h After Radiofrequency Ablation

The Doppler alterations before and 24 h after RFA in the TRAPs group are presented in Table 3. An increasing trend in the MCA-PSV and MCA-PI was observed in the pump fetuses before surgery compared with the controls (*P* = 0.048 and 0.687, respectively). After surgery, the MCA-PSV and MCA-PI of the pump fetuses increased further and showed significantly higher values than those of the controls (all *P* < 0.05), but there were no significant differences in the MCA-PSV and MCA-PI between the fetuses before and after surgery (all *P* ≥ 0.05). When all fetuses were divided into two groups, namely, GA of <20 weeks and GA of ≥20 weeks, this increasing trend (all *P* ≥ 0.05) and further increase (all *P* < 0.05) in the MCA-PSV and MCA-PI after surgery were still observed in the pump fetuses with a GA of ≥20 weeks, even when all Doppler values were corrected for by GA (expressed as Z scores) (all *P* < 0.05); however, such changes were not observed in those fetuses with a GA of <20 weeks (all *P* ≥ 0.05). There were no significant differences in the UA-PI or CPR among the three groups, whether these values were evaluated at a GA of ≥20 weeks or a GA of <20 weeks (all *P* ≥ 0.05).

**TABLE 3:** Doppler values in the normal control fetuses and pump fetuses, before and 24 h after RFA surgery.

Parameters	GA at doppler evaluation	Control	Presurgery	Postsurgery	Control vs. Presurgery	Control vs. Postsurgery	Pre- vs. Postsurgery	All *P*-value
UA-PI	15 W∼25 W (*n* = 28)	1.28 (0.42)	1.40 (0.39)	1.43 (0.48)				0.623
	<20 W (*n* = 12)	1.51 (0.28)	1.56 (0.30)	1.53 (0.14)				0.862
	≥20 W (*n* = 16)	1.189 (0.25)	1.27 (0.35)	1.15 (0.50)				0.214
UA-PI Z score	≥20 W (*n* = 16)	−0.197 ± 0.73	0.63 ± 1.23	0.34 ± 1.28				0.174
MCA-PSV(cm/s)	15 W∼25 W (*n* = 28)	24.79 (5.10)	30.75 (11.46)	30.76 (9.62)	0.048	0.015	1.000	0.010
	<20 W (*n* = 12)	25.21 ± 4.92	28.02 ± 8.94	28.16 ± 8.56				0.368
	≥20 W (*n* = 16)	24.79 (2.98)	32.45 (14.15)	35.64 (15.02)	0.102	0.024	1.000	0.019
MCA-PSV Z score	≥20 W (*n* = 16)	−0.49 ± 0.50	0.90 ± 1.83	1.39 ± 1.85	0.065	0.040	1.000	0.022
MCA-PI	15 W∼25 W (*n* = 28)	1.47 ± 0.20	1.54 ± 0.28	1.64 ± 0.36	0.687	0.015	0.326	0.018
	<20 W (*n* = 12)	1.48 ± 0.20	1.53 ± 0.28	1.58 ± 0.25				0.273
	≥20 W (*n* = 16)	1.44 (0.23)	1.47 (0.47)	1.72 (0.38)	0.279	0.031	1.000	0.032
MCA-PI Z score	≥20 W (*n* = 16)	−0.58 ± 0.84	−0.18 ± 1.20	0.37 ± 1.25	0.231	0.040	1.000	0.039
CPR	15 W∼25 W (*n* = 28)	1.13 (0.44)	1.09 (0.36)	1.16 (0.42)				0.069
	<20 W (*n* = 12)	0.92 (0.28)	0.99 (0.20)	1.03 (0.19)				0.205
	≥20 W (*n* = 16)	1.23 ± 0.31	1.22 ± 0.33	1.37 ± 0.24				0.305
CPR Z score	≥20 W (*n* = 16)	−0.30 ± 0.73	−0.63 ± 1.37	−0.01 ± 0.98				0.368

*RFA, radiofrequency ablation; UA-PI, umbilical artery pulsatility index; MCA-PSV, middle cerebral artery peak systolic velocity; MCA-PI, middle cerebral artery pulsatility index; CPR, cerebroplacental ratio; GA, gestational age; W, weeks. Values are given as the means ± standard deviations or as medians (interquartile ranges).*

## Discussion

In this study, we found an increasing trend in the MCA-PSV and MCA-PI of pump fetuses with a GA ≥ 20 weeks and a further increase after RFA surgery for TRAPs. However, such changes were not observed in those with a GA of <20 weeks. In addition, the UA-PI and CPR were not different between controls and pump fetuses before and after surgery. To our knowledge, this is the first study to assess alterations in the UA-PI, MCA-PSV, MCA-PI, and CPR of the pump fetus before and 24 h after RFA surgery.

In this study, although most of the pump fetuses showed no obvious signs of heart failure before surgery, they showed an increasing trend in the MCA-PSV. This increasing trend may be attributed to the high cardiac output of the pump fetus. According to previous reports, approximately 30% of the survival pump twins have congestive heart failure in the middle and late trimesters if untreated (9). In our study, only 2 (7%) pump fetuses had signs of congestive heart failure before surgery. This may be explained by the fact that the average GA assessed was relatively low, and the sizes of the acardiac fetuses were small; thus, the pump fetuses did not show obvious signs of heart failure. In our study, the pump fetus had an increasing trend in cerebral blood flow velocity before significant signs of heart failure, suggesting that the MCA-PSV appears to be an underlying hyperdynamic circulation of the pump twin.

In this study, the MCA-PSV of the pump fetus increased further after RFA surgery and showed a significantly higher value than that of the normal fetus. The relevant pathophysiological mechanism is still unclear. This further increase may be explained by two pathophysiological changes: congestive heart failure and hemodilutional anemia. In TRAPs, it is reasonable to assume that congestive heart failure may occur in pump fetuses after ablation surgery by receiving the blood that was supposed to go to the acardiac fetus. According to one new study, the occlusive procedure could aggravate the overworked heart, leading to heart failure (22), which is consistent with our findings. In our study, one fetus developed secondary pericardial effusion, suggesting that the operation may induce congestive heart failure. Previous studies have shown that fetal congestive heart failure is associated with increased blood flow velocities in the cerebral artery (11, 23). This might help in understanding why the pump fetuses showed increased MCA-PSV after surgery in our study. Hemodilutional anemia is a phenomenon that often occurs in heart surgery or surgery that involves heavy bleeding during extracorporeal circulation (24). Dilution of blood, as well as the accompanying decrease in viscosity, could contribute to the dilation of the cerebrovasculature, and an increase in cerebral blood flow (25, 26). After blocking blood to the acardiac fetus, the increase in hemoglobin might not match the increase in blood volume in the pump fetus in a short period. This insufficient increase in hemoglobin in a short period is very similar to the mechanism of hemodilutional anemia. Nevertheless, our surgery did not result in a dramatic increase in cerebrovascular velocity in the pump fetuses. This mild increase may be related to some autoregulatory capacity in fetal brains, but further studies are needed to address these speculations (27, 28).

In our study, the UA-PI and CPR were not altered either before surgery or after surgery, suggesting that the CPR may not be the best indicator for fetal hypoxia in those cases, and that the UA-PI cannot directly measure placental resistance in TRAPs because much of the blood is directed to the acardiac twin instead of passing through the placenta. In other words, our current results cannot show whether pump fetuses are hypoxic in this condition. Our results were not the same as the results reported by Peyvandi S et al., who found that pump fetuses (*n* = 19) had a lower MCA-PI than controls, suggestive of lower cerebral vascular impedance, and that the MCA-PI appeared to increase in a small group of pump fetuses (*n* = 6) after intervention (28). In our study, the increasing trend in the MCA-PI and the further increase after surgery may be due to the increase in the MCA-PSV rather than a “brain-sparing” effect. The unchanged UA-PI and CPR, before and after surgery, in pump fetuses further support this “non-brain-sparing” effect. In the future, new noninvasive indicators are urgently needed to investigate whether the pump fetus is hypoxic or whether placental resistance is increased in TRAPs.

In this study, no differences in RFA cycles, duration of coagulation or total operative time were observed between fetuses with a GA ≥ 20 weeks and fetuses with a GA of <20 weeks, suggesting that the Doppler alterations may be related to the hemodynamic changes caused by surgery, but it is unlikely to be related to the specific surgical procedures. However, there is still a lack of relevant reports, so this is worthy of further study.

Our findings have several clinical implications. On the one hand, fetal Doppler parameters, especially MCA-PSV, can sensitively assess fetal hemodynamic alterations and potential congestive heart failure. However, UA-PI and CPR cannot be used as valid indicators of placental resistance and fetal hypoxia in TRAPs. On the other hand, our findings provide a theoretical basis for the timing of treatment. At present, the optimal timing of therapy for TRAPs is controversial (29–32). For example, intrauterine therapies performed after 16 weeks can result in a live birth rate of 92%, but during the wait for intrauterine treatment, the death rate among pump fetuses is above 1/3 (29). However, if the intrauterine therapies are performed earlier than 13 weeks of GA, fetal loss increases to 41.7% (32). In our study, the increasing trend in the MCA-PSV before surgery and further increase after surgery were present in pump fetuses with a GA of ≥20 weeks, rather than a GA of <20 weeks, suggesting that treatment should be given before an underlying congestive heart failure occurs. In addition, it can avoid unnecessary acute hemodynamic changes associated with congestive heart failure after surgery.

Several limitations of our study should be mentioned. First, we cannot avoid the inherent risk of selection bias because of the retrospective design. Second, because of the rarity of this complication, the sample size was relatively small. Third, serial evaluations of fetal Doppler were hampered, as most patients were referred to our hospital, a tertiary referral center, and returned to local hospitals for subsequent examinations. Fourth, it is well known that fetal Doppler might vary with GA (14). Due to a lack of relevant reference data on fetal Doppler before 20 weeks, we did not compute the Z scores in this subgroup. However, we calculated the Z scores in the subgroup with a GA ≥ 20 weeks and observed the same results. In addition, a matching design was carried out, which may eliminate the influence of GA on outcomes to a certain extent. Finally, there was operator bias, as the ablation procedure is very much operator dependent. However, the surgeon (SH) in our study had more than 10 years of experience in intrauterine treatment, which would minimize operational deviations as much as possible.

## Conclusion

In conclusion, we found an increasing trend in the MCA-PSV and MCA-PI before surgery and a further increase 24 h after RFA surgery for TRAPs in pump fetuses with a GA of ≥20 weeks, rather than a GA of <20 weeks, whereas the UA-PI and CPR were not altered. The findings of this study suggest that in the middle second trimester, the pump fetus might suffer from high cardiac output rather than hypoxemia before surgery and a possible congestive heart failure or hemodilutional anemia after surgery in a short period, which may provide some theoretical evidence in favor of early intervention rather than waiting until a more advanced GA. Additional studies on the cerebrovascular response to altered hemodynamic conditions are needed to further understand the cerebral autoregulatory capacity of the pump fetus.

## Data Availability Statement

The raw data supporting the conclusions of this article will be made available by the authors, without undue reservation.

## Ethics Statement

As this was a retrospective analysis of routinely collected anonymized clinical data, the local Ethics Committee confirmed that no ethical approval from the patients was necessary in accordance with the national regulations.

## Author Contributions

CT and HQ: conceptualization. LZ and HL: formal analysis. HQ and SH: resources. ZW: supervision. LZ: writing of the original draft. CT, PB, and MK: writing review. All authors contributed to the article and approved the submitted version.

## Conflict of Interest

The authors declare that the research was conducted in the absence of any commercial or financial relationships that could be construed as a potential conflict of interest.

## Publisher’s Note

All claims expressed in this article are solely those of the authors and do not necessarily represent those of their affiliated organizations, or those of the publisher, the editors and the reviewers. Any product that may be evaluated in this article, or claim that may be made by its manufacturer, is not guaranteed or endorsed by the publisher.

## Footnotes

1
https://fetalmedicine.org
